# 

**Published:** 1872-08

**Authors:** 


### INDEX OF SUBJEC S.

ORIGINAL COMMUNICATIONS.

On Functional and Semi-Organic Diseases of the Heart.................449
Fracture of Femur in an Infant.................458
Enlarged Prostate—Suppressed Urine—Remedy.................462
Correspondence.................465

FOREIGN BORRESPONDENCE.

The Use of "Ferrum C: ndens" in Diseased Joints.................466

EXTRACTS AND GLEANINGS.

Chronic Chills, 472; A Good Local Enæsthetic, 474; Asthma Cured by Rubbing, 475; Hooping Cough—Croton Chloral Hydrate, 476; Subcutaneous Injection of Morphia and Chloroform -Iodine Salts in Syphilis, 477; Value of Salt—Pill for Constipation, 478; To Prevent Pitting from Small Pox, 479; Cold Food for Infants—Thoracentesis, 480; Hydrarthrosis Treated by Injection of Tincture of Iodine—Nocturnal Erections, 481; Hysteric Neuralgia—Commonly Called Spinal Irritation, 482; Cure for Colds—Chinese Treatment of Te'anus, 483; Muriate of Ammonia in Bronchitis, Catahrral Pneumonia. Etc.—Glycerinized Cotton for Dressing Wounds, 484; Cyanosis from Citrate of Silver, 485; Chloral in the Cure of Venereal Ulcers, 487; Nitro Muriatic Acid as an Hepatic Stimulant 487; Pinus Ganadensis, 488; Ergot in Paral sis of the Bladder, 489; Cerebro-Spinal Meningitis, 491; Hospital Gangrene and its Treatment with Camphor Gum, 492; Granular Lids, 493; Chloral in Certain Diseases of the Chest, 494; Opening Bubo with Caustic Potash, 495; Treatment of Gonorrhœa by Warm Water Injections—Tympanitis 496.

EDITORIALS, REVIEWS, ETC.

Notes on Our Visits to Colleges and Hospitals in Philadelphia.................498
Kind Words from Friends, with Comments.................503
Bibliographical.................508
Advertising Department.................512

